# k-SLAM: accurate and ultra-fast taxonomic classification and gene identification for large metagenomic data sets

**DOI:** 10.1093/nar/gkw1248

**Published:** 2016-12-13

**Authors:** David Ainsworth, Michael J.E. Sternberg, Come Raczy, Sarah A. Butcher

**Affiliations:** 1 Centre for Integrative Systems Biology and Bioinformatics, Division of Molecular Biosciences, Faculty of Natural Sciences, Imperial College London, SW7 2AZ, London, UK; 2 Illumina Inc., 5200 Illumina Way, San Diego, CA 92122, USA; 3 Bioinformatics Data Science Group, Department of Surgery and Cancer, Faculty of Medicine, Imperial College London, SW7 2AZ, London, UK

## Abstract

k-SLAM is a highly efficient algorithm for the characterization of metagenomic data. Unlike other ultra-fast metagenomic classifiers, full sequence alignment is performed allowing for gene identification and variant calling in addition to accurate taxonomic classification. A *k*-mer based method provides greater taxonomic accuracy than other classifiers and a three orders of magnitude speed increase over alignment based approaches. The use of alignments to find variants and genes along with their taxonomic origins enables novel strains to be characterized. k-SLAM's speed allows a full taxonomic classification and gene identification to be tractable on modern large data sets. A pseudo-assembly method is used to increase classification accuracy by up to 40% for species which have high sequence homology within their genus.

## INTRODUCTION

Metagenomics, the study of DNA extracted directly from microbial communities has been revolutionized by whole-genome shotgun sequencing. The ability to sample billions of short DNA reads from bacterial, viral and fungal species allows a unique insight into the taxonomic composition of diverse ecosystems as well as the processes taking place within. Metagenomic techniques have found applications in many areas, from ecological studies of acid mine drainage systems (1), soils (2) and oceans (3) to medical research involving communities of bacteria living within the human body (4). Human microbiome metagenomics aims to characterize the internal microbiota of healthy (5) and diseased individuals (6) and has been used to study obesity (7) and inflammatory bowel disease (8).

Currently, high-throughput sequencing technologies, e.g. Illumina's HiSeq or Life Technologies’ Ion Torrent are used to generate a whole-genome shotgun data set consisting of large numbers of short reads randomly sampled from the genomes of the species present in the sample. Computational methods are then used to assemble, assign taxonomies, or infer genes from the reads.

For taxonomic classification of reads, the speed of the computational methods has always lagged behind the rate of data generation and this has proved to be a large barrier to the widespread adoption of whole-genome shotgun based metagenomics. There are currently three main approaches to the taxonomic analysis of these data sets; sequence homology, composition based inference through machine learning and abundance estimation.

Homology based methods aim to find nucleotide sequences that are common to both the reads and a database and use these to infer taxonomy. Homology methods generally use pairwise alignment, where reads are compared to genomes to find sections where the sequences match. By finding the genomes that each reads maps to, taxonomy can be inferred. The alignment position along the genome can be used to identify the gene from which the read most likely originated as well as any variants between the read and the reference sequence.

The oldest and most common method is BLAST (9), which aligns sequences to a database of genomes and assigns taxonomy based on the best match. BLAST and other alignment based methods have high accuracy when the sampled species exists in the genome database but struggle to infer taxonomy otherwise. The vast size of the data sets, often more than 1 × 10^7^ reads means that these methods are incredibly slow as they have to align each read to thousands of large genomes. A BLASTN based analysis of a typical metagenomic data set can take weeks of compute time. Newer homology based methods such as Kraken (10) and CLARK (11) dispense with actual alignments, just using matching short *k*-mers to assign taxonomy. This allows greatly increased speed but does not produce actual alignments and therefore cannot identify genes, variants and alignment positions.

Composition based methods use machine leaning techniques such as Interpolated Markov Models (Phymm (12)) or Bayesian classifiers (NBC (13)) to extract sequence features. These methods have the advantage of not relying on a database (although one is needed to train the algorithm) but are less accurate than alignment based methods. Some methods, e.g. PhymmBL (12) and RITA (14), combine composition and homology based methods to improve accuracy.

Abundance based methods such as MetaPhlAn (15) use alignment to a greatly reduced database of specific genes to increase speed of analysis and produce a report of the relative abundances of species present in the sample. This however has the disadvantage of only aligning a subset of reads, preventing it from being used for pre-assembly binning or gene calling.

In this article we propose a novel metagenomic algorithm, k-SLAM (*k*-mer Sorted List Alignment and Metagenomics) which aims to bring together the advantages of all of the above methods whilst providing ultrafast run times. k-SLAM uses a *k*-mer method to rapidly produce alignments of the reads against a database and can therefore find genes and variants. A novel pseudo-assembly technique chains neighboring alignments together to improve taxonomic specificity. The main data structure is a sorted list of *k*-mers which makes k-SLAM extremely fast and parallelizable.

We report tests of k-SLAM against a variety of metagenomes composed of real reads and of simulated reads and compare the taxonomic accuracy and speed to five of the most common metagenomic algorithms; Kraken, CLARK, RITA, NBC and PhymmBL. k-SLAM is shown to be more accurate than all other current methods as well as being several orders of magnitude faster than both alignment based and composition based classifiers. We then validate k-SLAM against a variety of microbiome and environmental samples. Finally, we demonstrate a use case for k-SLAM's gene identification, a replication of the Rohde *et al.* crowd-sourced analysis of the Shiga toxin producing *Escherichia coli* O104:H4 (16). k-SLAM is shown to reproduce all of the main findings of the study, including determining various antibiotic resistance and toxin producing genes as well as their taxonomic origins.

## MATERIALS AND METHODS

### Algorithm outline

k-SLAM is a metagenomic classifier that uses a sequence alignment method to infer taxonomy and identify genes. Reads and database genomes are split into short (*k* = 32) *k*-mers which are added to a list and sorted so that identical *k*-mers are placed next to one another. Iteration through the list allows *k* base overlaps between reads and genomes to be found, along with alignment position. The overlaps are then verified with a full Smith-Waterman pairwise sequence alignment. Neighboring alignments are chained together along each genome into a ‘pseudo-assembly’, this allows reads that map to low complexity and conserved regions to still be classified precisely as the chains often extend into unique sequence. Low scoring alignments are screened and taxonomy is inferred from the lowest common taxonomic ancestor of the valid overlaps. Alignments are also used to infer genes and variants.

The sorted-list method of finding *k*-mer overlaps allows great speed and efficient parallelisation on modern hardware.

### *k*-mer based alignment

For the following analysis assume: *k* is an integer chosen at compile time (default *k* = 32) and a *k*-mer is a sequence of *k* nucleotides.

Each read is split up into overlapping *k*-mers (*k* − 1 base overlap) and the *k*-mers are added to a list. Each genome is split into non-overlapping *k*-mers (to save memory) and the *k*-mers are added to the same list. The list is sorted lexicographically, placing identical *k*-mers next to one another. The list is iterated over, finding overlaps between reads and genomes. For each of the overlaps found, a full Smith-Waterman pairwise local sequence alignment (using Mengyao Zhao's SIMD Smith-Waterman implementation (17)) is performed to ensure the overlap is valid and to find any variants. Alignments with a score lower than a user-chosen cutoff are screened (see Supplementary File 3: Supplementary Note 1 for a graph of sensitivity and specificity for various score cutoffs).

*k*-mers are stored along with their offsets from the start of the sequence, the identifier of the sequence from which they were extracted and a flag that is set if the *k*-mer has been reverse-complemented.

In order to find overlaps on both strands, k-SLAM compares each *k*-mer with its reverse complement and stores only the lexicographically smallest to save memory.

A similar *k*-mer based method using lexicographic sorting and spaced *k*-mer seeds, albeit for protein alignments, was independently discovered and used in DIAMOND (18).

### Paired-end reads

As read length is very important for taxonomic specificity, k-SLAM is designed to work with paired end reads of any insert size. Paired reads are treated initially as two single reads, which have their overlaps and alignments found using the above *k*-mer method. As bacterial sequence is often repetitive, it is highly likely that each end of the paired read aligns to multiple places on the same genome, hence a method is needed for detecting which pairings of these alignments are valid.

For each read/genome pair, all of the alignments are sorted by offset from the start of the genome. The algorithm then makes alignment pairs from each R1's nearest neighbor R2s and vice-versa.

This allows only a small subset of pairs to be considered instead of working with all possible pairs (avoiding *N*^2^ scaling). The library insert size is then inferred using a statistical method; insert sizes in the range of 0 ≤ *I* ≤ *Q*_3_ + 2(*Q*_3_ − *Q*_1_) are used to calculate a mean and standard deviation and all pairs with insert sizes *I* ≥ μ + 6σ are screened.

### Sequencing technologies

k-SLAM is designed to work with data from all of the most used sequencing technologies. There are, however, some constraints on the reads that affect accuracy. The reads need to be longer than the length of the *k*-mer and have a sufficiently low error rate such that there is at least one error free *k*-mer in each read. This allows a *k* base overlap to be found that can then be verified with a full Smith–Waterman alignment. Longer reads will produce more alignments and greater taxonomic specificity. Taxonomic specificity is also improved by using paired-end reads. A lower error rate allows a longer *k*-mer and hence a shorter compute time. k-SLAM has been tested and found to be accurate on Illumina HiSeq and MiSeq platforms as well as 454 and Ion Torrent data.

### Pseudo-assembly

Due to the similarity between the genomes of different bacterial species, there is a large probability that each read will map to more than one genome, this makes inferring taxonomy difficult as reads often map to long sections of conserved sequence.

k-SLAM attempts to solve this problem by grouping reads that map to adjacent locations on the same genome together into ‘pseudo-assemblies’. A new alignment score is calculated for each chain, taking into account per base similarity, chain length and depth of coverage. These long chains of reads often extend beyond conserved sections and into regions specific to one particular strain. This allows all reads within the chain to be assigned to the lowest possible taxonomic level. Following is a description of the k-SLAM pseudo-assembly algorithm as applied to each genome:

1. For each genome, sort the alignments by start position.
2. Form chains of alignments that overlap by more than 20 bases.
3. For each chain, calculate the following parameters:
  \begin{equation*} L=b_{\rm e}-b_{\rm s} \end{equation*}
  where *L* is the chain length (in nucleotides), *b*_s_ and *b*_e_ are the positions of the first and final nucleotides, respectively.
  \begin{equation*} C=N_{\rm b}/L \end{equation*}
  where *C* is the coverage and *N*_b_ is the sum of the number of bases in each read.
  \begin{equation*} \mu _{\rm s}=\frac{\Sigma {\rm s}}{N_{\rm b}} \end{equation*}
  where *μ*_s_ is mean score per nucleotide and *s* is the Smith–Waterman score for each alignment.
  \begin{equation*} S=C \mu _{\rm s} L \end{equation*}
  where *S* is the chain's score which is applied to all of the alignments in the chain.

### Inferring taxonomy

k-SLAM infers taxonomy using a lowest common ancestor technique similar to that in the Huson *et al.* program MEGAN (19). For each read, a score cutoff is calculated by multiplying the highest alignment score by a user chosen constant and all alignments below this cutoff are screened (see Supplementary File 3: Supplementary Note 2 for a graph of sensitivity and specificity for various fractional score cutoffs). Taxonomy is chosen based on the lowest common ancestor in the taxonomy tree of the remaining alignments. A matching gene is also inferred for each read from the position of the alignment along the genome.

### Inferring genes

Genes are inferred using the GenBank format annotations. For each non-screened alignment, the gene with the most overlapping bases is chosen. For the XML output, genes with identical names, protein IDs or products that are assigned to the same taxonomy are combined into a single entry with an associated count.

### Synthetic metagenomes

In order to calculate the taxonomic classification accuracy, a data set is required for which every read's taxonomic origin is known. This is impossible for real environmental metagenomes so the standard procedure is to produce these data sets *in silico* (20, 21). The first testing data set was entirely artificial, using paired-end 150 bp reads generated from 100 randomly selected NCBI genomes with an error profile five times greater than would be expected from Illumina reads (this error profile was taken from the Kraken (10) paper). This data set, which has all species in equal proportions, was designed so that it can be seen how the accuracy of each classifier changes for different species. The second data set was taken from Metabenchmark (22), an effort to evaluate the accuracy and speed of metagenome analysis tools. The data set used was >50 million reads and designed to accurately mimic the complexity, size and characteristics of real data. The researchers created the data sets by sampling read pairs from sequenced genomes in well defined proportions with error profiles added from real HiSeq data.

## RESULTS

### Taxonomic classification accuracy

In order to compare the accuracy of k-SLAM's taxonomic assignment with other tools, each of the two test data sets were analyzed using Kraken, CLARK, PhymmBL, NBC and RITA (all using the RefSeq bacterial genomes database) and the number of reads correctly assigned per-species was calculated.

For the first data set, k-SLAM classified more reads successfully than any other program. A total of 96% of reads were assigned correctly at the species level versus 84% for RITA, 91% for CLARK, 92% for Kraken, 94% for PhymmBL and 94% for NBC.

**Figure 1.**
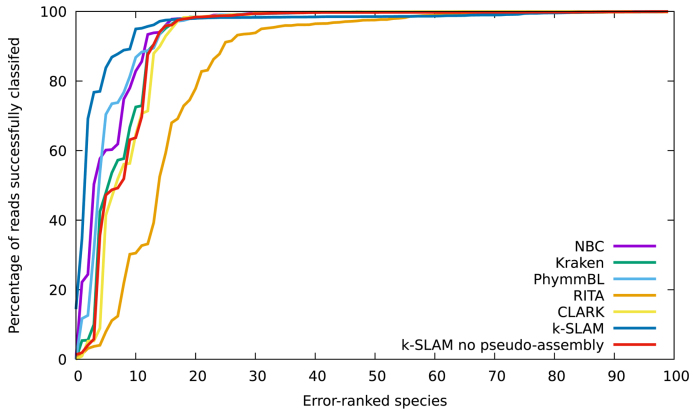
Taxonomic classification accuracy comparison for an artificially generated data set consisting of 100 randomly selected species. k-SLAM classified 96% of reads successfully versus 84–94% for other programs. Pseudo-assembly increases accuracy from 92% to 96%. There are 17 species which are classified poorly for all programs (see Figure 2).

**Figure 2.**
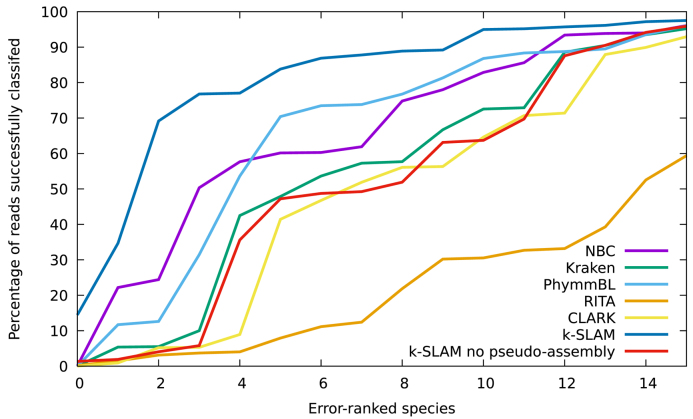
Enlargement of Figure 1 showing only the 15 worst classified species. k-SLAM is far more accurate on these species due to pseudo-assembled contigs extending beyond conserved regions.

In order to determine which species were classified best by each program, a graph of per-species accuracy was plotted (see Figures 1 and 2).

Although k-SLAM's accuracy is consistently high across the 100 species, it can be seen that it is particularly accurate for the 20% of species that the other classifiers struggled most with. These difficult species were examined and it was found that there were 17 bacteria that appeared in the bottom 25 species of all classifiers (excluding k-SLAM) (see Supplementary File 1: Supplementary Table S1). These species often have high genus accuracy (>95%) but low species accuracy (often <50%), suggesting that they share large regions of their genomes with other species in their genus. Any read mapped within a conserved region can therefore only be assigned at the genus level. k-SLAM circumvents this issue using pseudo-assembly, where overlapping alignments can be chained together to give a contig that extends beyond the conserved region into unique sequence. The advantage of pseudo-assembly can be seen from Figures 1 and 2; improving the classification accuracy on difficult species above that of other methods (41% more reads classified than Kraken) and increasing the total number of reads successfully classified from 92% to 96%.

**Figure 3.**
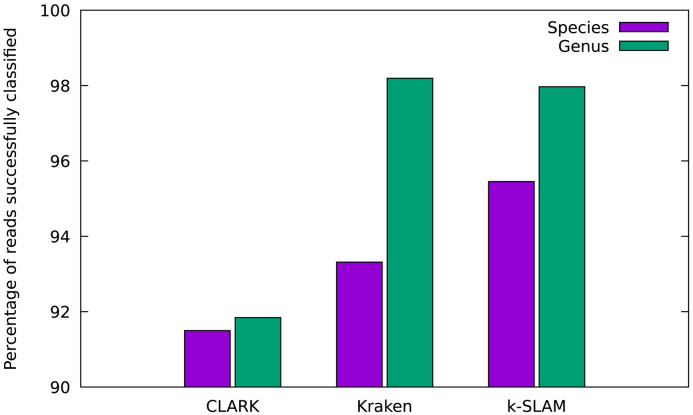
Comparison of species and genus classification accuracy for the bacterial/archaeal species from the Metabenchmark (22) data set. k-SLAM classified 96% of reads successfully at species level versus 91–93% for other programs. The small difference between species and genus accuracy for k-SLAM (compared to Kraken) is due to the increased specificity of taxonomic assignments given by k-SLAM's pseudo-assembly.

In order to determine which classifier performs best on a sample with a more realistic distribution of species, the Metabenchmark data set was analyzed using the three fastest classifiers (k-SLAM, Kraken and CLARK). A graph of species and genus classification accuracy for each program was plotted (see Figure 3). It can be seen that k-SLAM has the greatest species level accuracy across the three classifiers. Whilst k-SLAM obtained marginally fewer alignments than Kraken, it assigned taxonomy more specifically, this can be seen by the much smaller gap between genus and species accuracy. As with the first data set, this increase in specificity is due to pseudo-assembly.

### Speed and computational requirements

**Figure 4.**
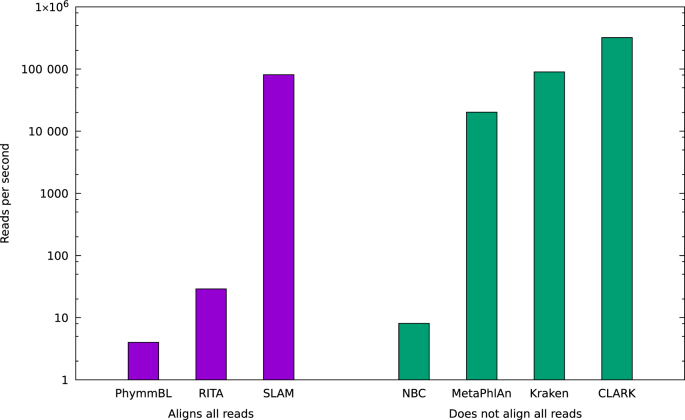
Speed comparison of classifiers. k-SLAM is at least 2800x times faster than other alignment based classifiers and comparable in speed to non-alignment based Kraken and CLARK. An improvement of several orders of magnitude allows gene and variant calling to be computationally tractable for large metagenomic data sets.

With the size of metagenomic data sets rapidly increasing, the speed of taxonomic assignment algorithms has become more important. To compare classification speed, each of the different programs were used to analyze the Human Microbiome Project SRS011061 data set (from the NCBI Sequence Read Archive). In order to better recreate modern computational environments, the speed on eight CPU cores (Xeon E7-8837 2.67GHz) was measured (see Figure 4) and to ensure a consistent test, only single end reads were used and the database read time was not counted.

When comparing speed, it is important to distinguish between different types of classification algorithm. Programs such as PhymmBL and RITA that align all reads to a database tend to be many orders of magnitude slower than methods such as Kraken and CLARK (that use *k*-mer based classifiers) or MetaPhlAn (that aligns only a fraction of the reads to a small database). The alignment based methods, however, have the advantage of knowing exactly where each read mapped to a specific genome. This can be used to extract other important information such as overlapping genes and sequence variants.

Compared to the other classifiers that use sequence alignment, k-SLAM's speed of 81 000 reads per second was 2800x faster than RITA, 10 000x faster than NBC and 20 000x faster than PhymmBL. k-SLAM was slightly slower than Kraken and 4x slower than CLARK, but in addition to taxonomic classification, real alignments were generated. This greatly increased speed (compared to other alignment based methods) allows gene and variant calling to be computationally viable for modern large metagenomic data sets. The speed increase of k-SLAM is due to a *k*-mer based alignment using a list-sort to find identical *k*-mers. This is very fast and parallelizable on modern hardware. For a fixed database size and *N* reads, the execution time scales with *N*log *N* and the memory usage with *N*. The disadvantage of this sorted list method, however, is large memory usage due to all reads being analyzed simultaneously. It is for this reason that data sets can be split by k-SLAM into smaller subsets of 1–10 M reads before analysis, allowing an upper limit of memory to be set. k-SLAM uses around 50 GB RAM when analyzing data in 10 million paired read subsets.

### Validation using real metagenomes

**Figure 5.**
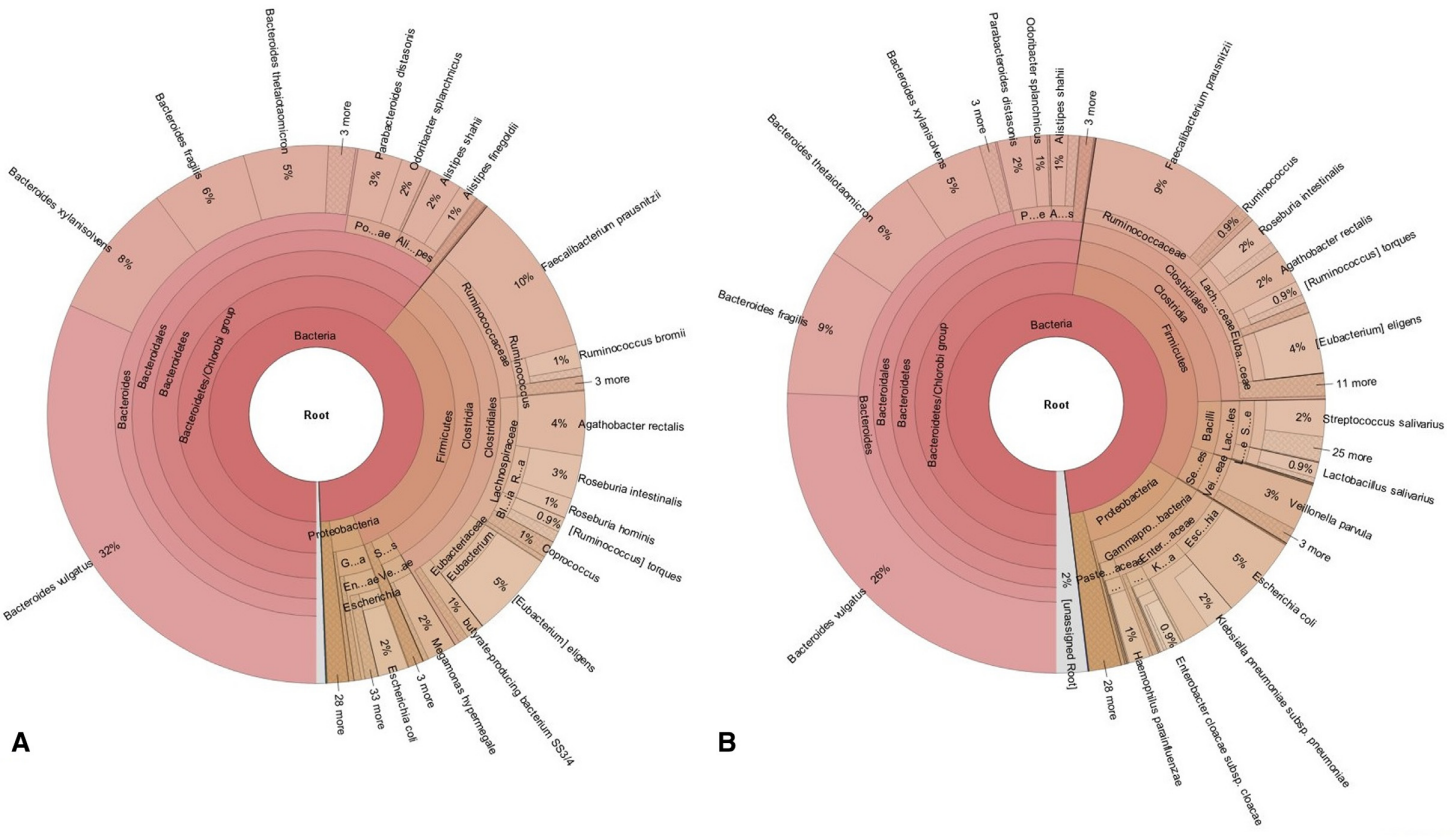
Analysis of liver cirrhosis data. Taxonomic distribution of gut microbiome reads. (**A**) Healthy individuals (**B**) Patients with liver cirrhosis. Around 30–40% of reads were assigned taxonomy.

In order to validate the algorithm, we re-analyzed the data from the Qin 2014 (23) study that compared gut microbiomes in healthy individuals and those with liver cirrhosis. The study found large differences in the gut flora between the two groups which suggested an invasion of the gut with oral commensals in those with cirrhosis. We used k-SLAM to analyze all 181 data sets against the NCBI bacterial genomes database (see Figure 5) and found several notable differences in gut microbial composition that confirm the results of the original study. Taxons that were enriched in healthy individuals include *Lachnospiraceae* (11% versus 6% of reads) and *Ruminococcaceae* (13% versus 11%) that provide gut protective effects. Taxons enriched in individuals with cirrhosis include *Veillonellaceae* (4% versus 2%) and *Streptococcus* (4% versus 0.5%) that contain species of oral origin known to cause opportunistic infections. In addition to the results from the study, we also found greatly increased amounts of *E. coli* (5% versus 2%) and *K. pneumoniae* (3% versus 0.5%) in patients with cirrhosis. In addition to the gut microbiome, an environmental sample (of inhalable microorganisms in air pollution) was analyzed (see Additional file 3: Additional note 3).

### Genomic analysis of Shiga toxin producing *E. coli*

In May and June, 2011, there was an outbreak of a foodborne strain of *E. coli* in Germany that infected over 3000 people and caused 40 deaths. In order to discover why this strain was so virulent, Rohde *et al.* sequenced an isolate of the pathogen using high-throughput bench top machines and made the data openly available online (16). Within a week, a large scale crowd-sourced analysis had revealed that the *E. coli* strain (O104:H4 str. TY-2482) had acquired genes for Shiga toxin 2 (via a prophage) and for resistance to several antibiotics.

At first glance it may appear that a metagenomic analysis of a single isolate would not provide any insight, however, the strain in question was novel and appeared to have acquired genes from bacterial and viral strains. If the sample was analyzed using one of the current purely taxonomic classifiers the results would show a mixture of false positive strains and could not reveal any of the genes shared with known species. k-SLAM on the other hand uses real alignments and can identify individual genes present in a sample (as long as they are in the genome database) along with their taxonomic origin and so could be useful in determining the genetic makeup of an unknown strain. This would allow the toxin producing genes to be identified, along with any antibiotic resistance genes, aiding treatment.

A database was constructed from all complete *E. coli* genomes that were available at the time of the original article, plus all NCBI bacterial and viral genomes (with *E. coli* removed). k-SLAM was used to identify the genes present in the outbreak strain genome, along with the inferred taxonomic origin for each gene (see Table 1).

**Table 1.** Some of the genes found by k-SLAM in the outbreak *E. coli* strain, including genes for Shiga toxin and for antibiotic and antibacterial resistance. All relevant genes (Shiga toxin, tetracycline resistance, streptomycin resistance and tellurite resistance) from the study were found along with their taxonomy. Genes for resistance to chloramphenicol, copper and polymyxin were found

**Genetic feature**	**Predicted taxonomic origin**
Shiga toxin 2 subunit A	*Escherichia* phage P13374
Shiga toxin 2 subunit B	*Escherichia* phage P13374
Tetracycline resistance protein *tetA*	*Escherichia coli*
Tellurite resistance	*Escherichia coli* 55989
Copper resistance	*Escherichia coli* 55989
Chloramphenicol resistance	*Escherichia coli* 55989
Streptomycin phosphotransferase	*Escherichia coli* O111:H- str. 11128
Polymyxin resistance protein B *pmrD*	*Escherichia coli*
Multidrug resistance *mdtN, marC, emrD, mdtQ*	*Escherichia coli* 55989
Multidrug resistance *norM, mdtH, ebrB, mdtK*	*Escherichia coli*

k-SLAM found alignments for 98.5% of the reads, with 2.42% of these alignments being viral strains and the rest bacterial. The predominant *E. coli* match was strain 55 989 with 47% of reads mapping unambiguously to it. Other contributing strains were E24377A with 7.3% and SE11 with 6.9%. This matches the results of the original paper that found a 99.84% nucleotide identity between the outbreak strain and 55 989.

k-SLAM identified all of the important genes from the study (Shiga toxin, tetracycline resistance, streptomycin resistance and tellurite resistance) along with their taxonomic origins. Additionally, genes for resistance to chloramphenicol, copper and polymyxin were found (see Table 1). Analysis took less than 10 min on 8 Xeon E7-8837 2.67 GHz cores, far less than the week taken for the original study.

## DISCUSSION

We have presented a metagenomic classifier that is several orders of magnitude faster than other alignment based algorithms and more accurate than any current classifier. The greatly increased speed, whilst still producing real alignments for every read against a database of genomes, allows gene and variant calling to be computationally viable with modern metagenomic data sets.

Across a variety of species, k-SLAM was shown to be between 12% and 2% more accurate than other classification methods. This increase in accuracy is particularly noticeable for species that have large sections of their genomes conserved within their genus. For these difficult species that comprised 20% of the first testing data set, k-SLAM can provide a significant (around 40%) increase in the number of reads assigned correctly. This increased accuracy was shown to be as a result of chaining adjacent alignments into a pseudo-assembly, extending beyond conserved sections of genomes. k-SLAM was shown to be 2800x faster than other alignment based classifiers and comparable in speed (within the same order of magnitude) to Kraken and CLARK, which do not produce alignments or gene identification.

k-SLAM's ability to identify genes in a sample of a novel isolate was demonstrated in the replication of the crowd sourced analysis of the Shiga toxin producing *E. coli* strain. All antibiotic resistance and toxin producing genes from the study, as well as their predicated taxonomic origins, were found.

Alignment based methods produce greater accuracy than composition based methods and also allow genes to be identified. They also assign taxonomy to all reads unlike abundance estimation methods. The disadvantage had always been that they were far slower. k-SLAM has been demonstrated to solve this problem, providing a speed improvement of several orders of magnitude over older alignment based algorithms. This makes alignment based metagenomics faster than abundance estimation, comparable in speed to pure taxonomic classifiers and more accurate than all existing methods.

The primary limitation of k-SLAM (and all other homology based classifiers) is that taxonomy cannot be predicted if a similar species does not already exist in the database. This problem is being mitigated by the recent rapid increase in size of genome databases, including strains assembled from mixed metagenomic samples. Another limitation is large memory usage, k-SLAM requires around 50 GB of RAM for an average metagenomic data set.

The analysis of the Shiga toxin producing *E. coli* sample showed that k-SLAM can be used to identify genes present in a novel strain as well as their taxonomic origin. This could be used as a rapid diagnostic procedure to detect antibiotic resistance and toxin producing genes to aid medical treatment. This can only be done with an alignment based classifier and k-SLAM makes this computationally tractable.

Finding individual alignment positions instead of purely taxonomic classification can aid with revealing false positive species as they will often only have reads mapping to a small subset of genes.

The taxonomic classification of a mixed data set indicates that k-SLAM could be used for binning prior to metagenomic assembly or to screen for contaminants in an isolate data set. A possible use case would be in a quality control step to identify contaminants in a single strain data set in order to find their origin and remove from further sequencing experiments. Another application would be to screen bacterial sequence from human saliva samples prior to mapping to the human genome. In human microbiome metagenomics, k-SLAM could be used to identify gut parasites (protozoa, nematodes and helminths) and also to detect plant sequence that can indicate dietary components. k-SLAM's increased speed makes this analysis feasible. The increased accuracy due to pseudo-assembly allows bacteria that have large sections of sequence conserved within their genus (as is the case with common intestinal flora) to still be classified accurately, aiding with pre-assembly binning. This type of analysis cannot be done with abundance estimation methods as they do not align all reads.

## AVAILABILITY

k-SLAM is available at github.com/aindj/k-SLAM and a web-server is provided at sbg.bio.ic.ac.uk/slam/.

## Supplementary Material

Supplementary Data
