# Supplementary material for: k-SLAM: accurate and ultra-fast taxonomic classification and gene identification for large metagenomic data sets
Source: Nucleic Acids Res. 2016 Dec 13;45(4):1649–56. doi: 10.1093/nar/gkw1248 (PMC5389551; doi:10.1093/nar/gkw1248)
Supplement: Supplementary Data [file gkw1248_Supp.zip › nar-01842-n-2016-File009.pdf]

Supplementary file 1.pdf - Contains supplementary table 1.

Supplementary table 1.csv - Contains raw data for supplementary table 1.

Supplementary file 3.pdf - Contains supplementary notes 1 and 2.
