# Supplementary material for: k-SLAM: accurate and ultra-fast taxonomic classification and gene identification for large metagenomic data sets
Source: Nucleic Acids Res. 2016 Dec 13;45(4):1649–56. doi: 10.1093/nar/gkw1248 (PMC5389551; doi:10.1093/nar/gkw1248)
Supplement: Supplementary Data [file gkw1248_Supp.zip › nar-01842-n-2016-File010.pdf]

(See supplementary table 1.csv for raw data)

Supplementary table 1: The 17 species which were amongst the worst classified 25 species for all classifiers. The genus accuracy is usually high but the species accuracy is low. k-SLAM provides a large increase in accuracy for these species (because of pseudo-assembly), successfully classifying on average 41% more reads than Kraken.

		CLARK		Kraken		NBC		PhymmBL		RITA		k-SLAM	
Strain	Accession	Sp.	Gen.	Sp.	Gen.	Sp.	Gen.	Sp.	Gen.	Sp.	Gen.	Sp.	Gen.
Serratia sp. AS12	NC_015566.1	0.027	2.918	0.3	99.8	0.5	99.7	31.5	99.7	0.2	11.3	14.5	99.7
Myc. tuberculosis CTRI-2	NC_017524.1	5.317	7.818	5.4	100	22.2	99.9	88.4	100	3.7	23.2	76.8	100
Myc. bovis BCG 1173P2	NC_008769.1	5.203	7.912	5.5	100	61.9	100	11.7	100	1.5	23.1	34.7	100
Myc. tuberculosis NITR206	NC_021194.1	8.932	11.4	10	100	24.4	99.9	88.8	100	4	23.3	77	100
Pse. putida S16	NC_015733.1	41.417	42.808	42.5	99.9	57.7	99.9	73.5	99.9	21.9	39.8	69.2	99.6
Ric. japonica YH	NC_016050.1	46.753	51.354	47.9	100	50.3	100	53.6	100	3.1	29.6	83.8	99.9
Myc. intracellulare MOTT-02	NC_016947.1	51.866	53.22	53.6	99.9	82.9	100	70.4	100	7.9	22.3	88	99.8
Bur. pseudomallei 1026b	NC_017831.1	56.329	58.339	57.3	100	78	99.8	76.8	99.9	32.7	69.9	89.2	99.5
Pec. wasabiae WPP163	NC_013421.1	56.08	57.291	57.7	99.7	93.4	99.7	73.8	99.8	11.1	15.8	86.9	99.3
Ric. massiliae MTU5	NC_009897.1	64.624	67.874	66.7	100	74.8	100	81.3	100	12.4	35.9	88.9	99.9
Tre. pallidum str. Gauthier	NC_016843.1	70.688	70.972	72.5	100	60.1	99.9	96.1	100	30.2	42.8	99.5	99.9
Tre. pallidum str. Nichols	NC_021490.2	71.39	71.696	72.9	100	60.3	100	96.2	100	30.5	42.5	99.7	100
Ana. centrale str. Israel	NC_013532.1	87.913	88.136	88.6	100	85.6	100	86.8	100	72.9	77.4	95.2	99.2
Cal. kronotskyensis 2002	NC_014720.1	89.923	90.466	90.5	100	93.9	100	89.5	99.9	33.2	45.9	95	99.4
Str. pyogenes HSC5	NC_021807.1	92.937	93.175	93.5	100	97.9	100	97.4	99.9	68	75.2	99.5	99.9
Kle. pneumoniae 1084	NC_018522.1	95.063	95.093	95.2	96.8	96.1	97.2	97.2	97.7	52.5	53.9	98.3	98.5
Bur. multivorans ATCC 17616	NC_010070.1	97.239	97.336	97.5	99.9	98	99.6	97.9	99.7	83.1	91.1	97.9	99.1
