# Supplementary material for: k-SLAM: accurate and ultra-fast taxonomic classification and gene identification for large metagenomic data sets
Source: Nucleic Acids Res. 2016 Dec 13;45(4):1649–56. doi: 10.1093/nar/gkw1248 (PMC5389551; doi:10.1093/nar/gkw1248)
Supplement: Supplementary Data [file gkw1248_Supp.zip › nar-01842-n-2016-File011.pdf]

# 1 Supplementary note 1

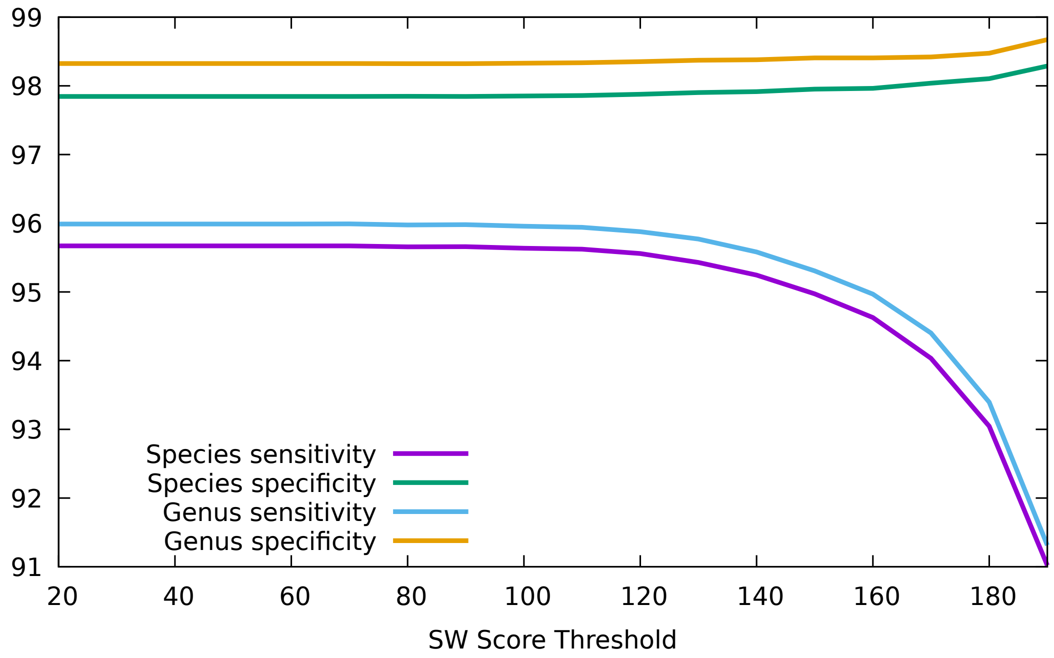


Figure 1: The variation in sensitivity and specificity for different Smith-Waterman alignment score cutoffs

# 2 Supplementary note 2

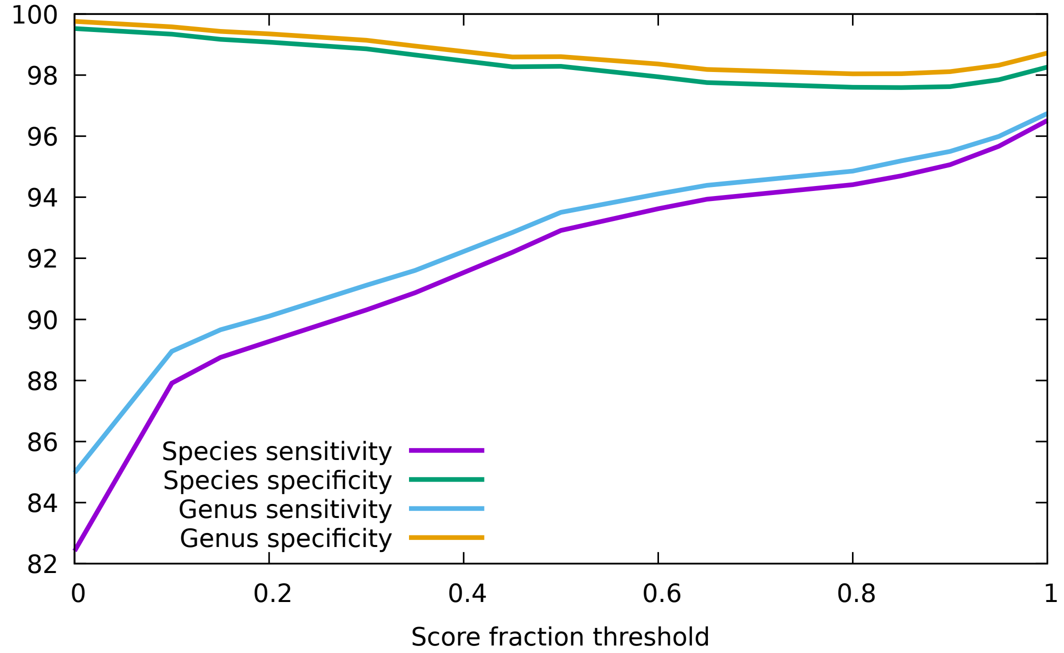


Figure 2: The variation in sensitivity and specificity for different fractional score cutoffs

# 3 Supplementary note 3

In order to validate the k-SLAM algorithm, an environmental sample was analysed. The sample chosen was from the Cao et al. 2014 study [1] where inhalable microorganisms in a severe smog event were analysed using high throughput whole metagenome sequencing. The results of k-SLAM's analysis corresponded directly to those from the original study (which used MetaPhlAn [2]), identifying all of the most abundant bacterial species.

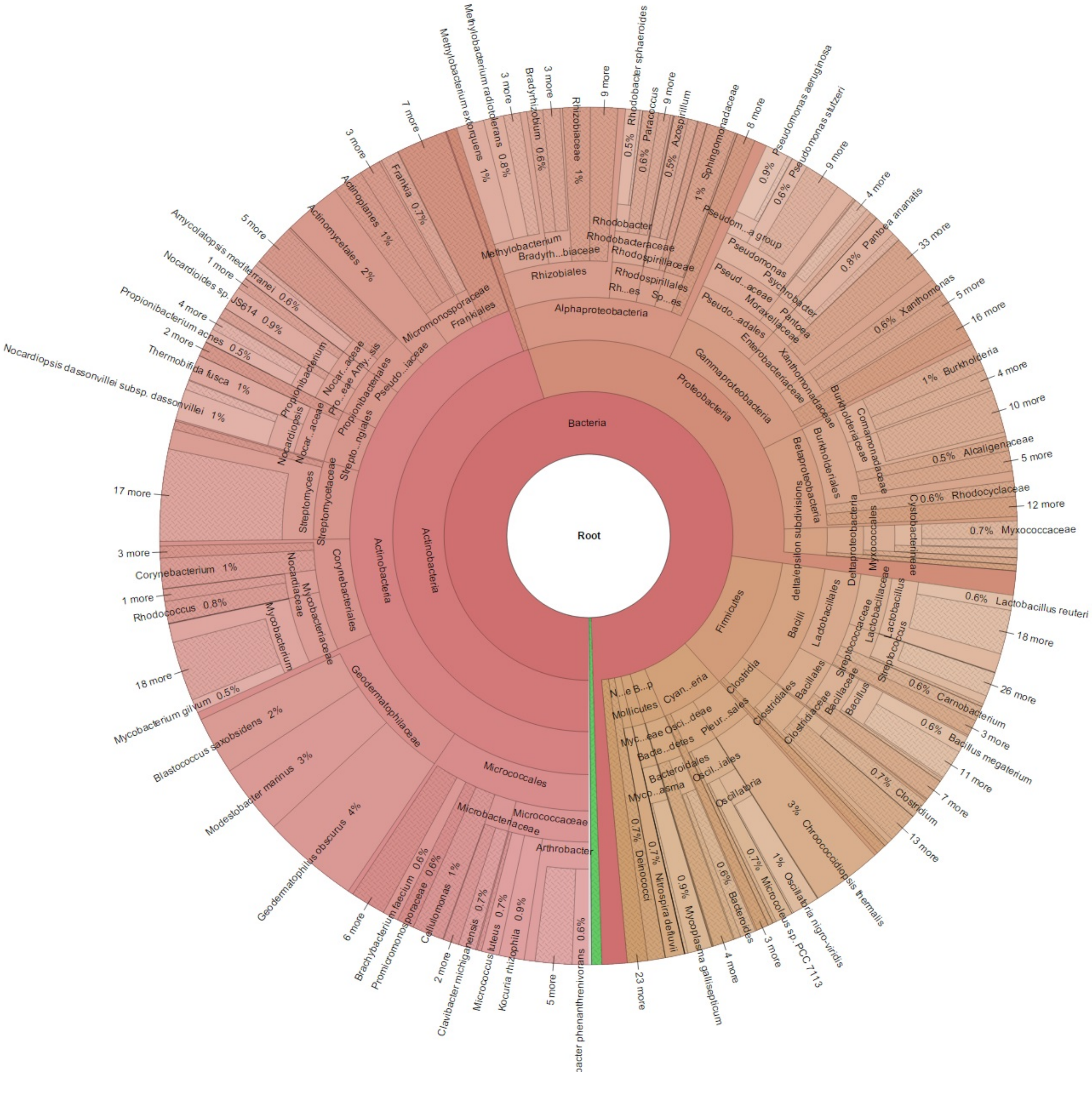


Figure 3: Taxonomic distribution of air pollution reads

# References


[1] Cao, C., Jiang, W., Wang, B., Fang, J., Lang, J., Tian, G., Jiang, J., Zhu, T.F.: Inhalable microorganisms in Beijings PM2. 5 and PM10 pollutants during a severe smog event. Environmental science & technology **48**(3), 1499–1507 (2014)

[2] Segata, N., Waldron, L., Ballarini, A., Narasimhan, V., Jousson, O., Huttenhower, C.: Metagenomic microbial community profiling using unique clade-specific marker genes. Nat. Methods **9**(8), 811–814 (2012)
